# Relationship between physical activity and mental health in women after childbirth: a cross-sectional exploratory study

**DOI:** 10.1186/s12884-022-04758-0

**Published:** 2022-05-23

**Authors:** Yumi Tomioka

**Affiliations:** Faculty of Nursing, Toho University, 4-16-20 Omori-nishi, Ota-ku, Tokyo, 143-0015 Japan

**Keywords:** Physical activity, Accelerometer, MET, GHQ-28, Mental health, 2 months postpartum, Housework, Child-rearing, WHO standard, Tokyo

## Abstract

**Background:**

Physical activity (PA) is recommended for women after childbirth. However, it is unknown whether PA, such as housework and child-rearing, is associated with mental health. This study aimed to measure daily PA in women 2 months postpartum as well as investigate the relationship between daily PA and mental health.

**Methods:**

In this cross-sectional quantitative exploratory study conducted between September 2017 and May 2018, 110 women were approached for participation. Mental health was evaluated using the General Health Questionnaire-28, and PA measurements were performed using accelerometers that the participants wore for 2 days. Welch’s *t*-test and linear regression analysis were performed to assess the relationship between PA and mental health.

**Results:**

This study included 99 participants. The mean amount of daily activities from housework and child-rearing was 3.21 ± 1.14 metabolic equivalent of tasks (METs)-h/day and that of time spent sitting was at least 7.5 h/12.5 h. PA time spent in light child-rearing and housework activities was significantly longer among multiparous women than among primiparous women (*t* =  − 3.41). PA time comprising the duration of moderate (3 METs) or more vigorous PA was 73 min/day. No significant relationship between mental health and PA was observed. However, the amount of daily activities tended to increase with an improvement in mental health. The amount of daily activities exceeded 3 METs-h/day regardless of the mental health status.

**Conclusions:**

No significant relationship was found between the amount of daily activities and mental health. The former increased as the latter improved. The amount of daily activities met the standard recommended by the World Health Organization, regardless of the mental health status.

## Background

The importance of physical activity (PA) in women after childbirth is widely known, which is why it is recommended. The World Health Organization (WHO) recommends moderate PA for 150 min/week for activities of daily living and housework [1]. This recommendation is viewed as an important part of health promotion in many countries worldwide [2–5]. The effects of postpartum PA include the maintenance and improvement of mental health in addition to physical recovery [6, 7]. However, many obstacles that prevent women from engaging in PA have been reported [8]. These obstacles include the lack of time [9–13], issues with child care [9], fatigue, and poor physical condition [9, 12], social isolation [11], being overwhelmed by motherhood responsibilities [11], lack of motivation [11, 12], lack of appropriate exercise facilities, and lack of professional advice on exercise [11].

To the best of my knowledge, the degree of PA from daily housework and child-rearing remains unknown. In addition, the relationship between unstable mental health and PA in women needs to be elucidated. Studies on postpartum PA can be of two types: self-reported studies and studies using objective measurements. Many studies are of the former type and a few are of the latter. Both the studies have reported PA time for each intensity and sedentary time [7, 10, 14–17]; some studies have explored the number of steps measured using accelerometers [14, 15, 18]. Both types of the abovementioned studies found that the participants spent a significant period doing *light* PA, indicating that they led a sedentary lifestyle. In terms of the association between PA and mental health, vigorous PA tends to improve mental health in pregnant and parturient women. In articular, continuous PA from pregnancy to postpartum attenuates the symptoms of postpartum depression and improves postpartum well-being and quality of life [19–27]. In contrast, another previous study showed that PA is not associated with chronic life stress [28]. These studies explored the relationship between PA and mental health. However, to the best of my knowledge, objectively measured PA (in terms of housework and child-rearing) status remains unknown and no studies have investigated the relationship between unstable mental health during the postpartum period and PA. Much of these women’s time is spent doing daily activities of living at home; thus, elucidating the amount of PA in terms of housework and child-rearing may be significant from the perspective of postpartum quality of life.

Therefore, the present study aimed to investigate PA (in terms of housework and child-rearing) performed by women at 2 months postpartum as well as the relationship between PA and mental health. The study outcomes may help provide information to women regarding specific and efficient PA during the postpartum period, which will facilitate recovery to their prepregnancy physical status and health improvement.

## Methods

### Study design

This was a cross-sectional quantitative exploratory study aimed at examining the relationship between PA and mental health.

### Participants

The participants were women at 2 months postpartum who were raising their children. These women were targeted because their reproductive organs, which had changed during pregnancy and childbirth, have returned to their prepregnancy state and they have resumed their activities of daily living (e.g., housework and child-rearing) in this period. The inclusion criteria were healthy women who had lived in A District of Tokyo since their pregnancy, had healthy infants, and could wear an accelerometer for 2 days. The exclusion criteria were women with any mental disease, those who were instructed to restrict PA by a physician, those who worked, those who did not do housework, or those who did aerobic exercise or strength training.

For convenience, the participants were selected from women who gave birth at a certain obstetrics clinic (one center) in A District of Tokyo.

As this was an exploratory study, it was difficult to determine the sample size [29]. Thus, Green’s formula (N ≥ 104 + m) was used to determine the sample size for regression analysis [30]. Therefore, 110 women were included. The number of individuals requested to participate was restricted to 110 because of the decreasing numbers of births at the participating center and restrictions during the study period as well as the number of measurement devices available for the study.

### Data collection method

#### Study period

The survey was conducted from September 2017 to April 2018.

#### Participation method

The participants were women at 2 months postpartum who were randomly selected during the study period. The participants were given an explanation about the objectives of the study, details of their participation, and methods to be used in the study. Written consent was obtained from those who agreed to participate. The participants were surveyed via mail. They were sent a questionnaire assessing participant characteristics, the General Health Questionnaire (GHQ)-28, an accelerometer, return envelopes, withdrawal forms, and documents describing the details of their participation and study methods. These materials were returned via mail after survey completion.

### Survey details

#### Questionnaire

GHQ-28 (Japanese-language version) was used to assess the mental health status of the participants. It is an abbreviated version of the GHQ that has been translated into Japanese. This scale was developed by Goldberg and Hillier [31] and was translated into Japanese by Nakagawa and Daibo [32]. It has been approved by the WHO as the best scale to assess anxiety disorders, and the validity and reliability of the Japanese version have been thoroughly verified [33–36]. The GHQ-28 comprises four subscales: somatic symptoms, anxiety and insomnia, social dysfunction, and depression. This questionnaire was scored on either a 2-point scale (not at all and no more than usual = 0 points and rather more than usual and much more than usual = 1 point, with a total score of ≥ 6 indicating that the respondent has mental health problems) or a Likert scale (not at all = 0 points, no more than usual = 1 point, rather more than usual = 2 points, and much more than usual = 3 points).

#### Measurement methods and data collection

PA measurements were performed using three-dimensional accelerometers. The participants were requested to wear the devices for 24 h during a period of 2 days, except when bathing and sleeping. The devices were strapped onto the participants’ waists using a belt.

The optimal period of PA measurements remains controversial. Some studies recommended approximately 1 week [14, 17], whereas others indicated that the results of a measurement period of 2–3 days were highly correlated with those of a measurement period of > 4 days [37–39]. In the pretest, continuous and appropriate wearing of the device was difficult for the participants, and at the same time, there was a high burden on the participants as it interfered with their child-rearing activities. Considering this, we used a PA measurement period of 2 days. The participants were instructed to appropriately wear the device for 2 days only on usual weekdays.

We used Active Style Pro HJA-750C accelerometers (Active Style Pro HJA-750C: OMRON, OMRON Healthcare, Kyoto, Japan). The device detects PA from activities, such as walking, housework, and child-rearing every 10 s using three-dimensional speed acceleration sensors (accelerometer measurements in three directions on the X-, Y, and Z-axes) and automatically calculates the intensity of PA (metabolic equivalent of task [MET]) as well as the amount of PA (MET-h). The device stores the data for 45 days. Further, 1 MET is equivalent to the oxygen intake of 3.5 mL/kg of body weight/min in a resting (sitting) position. The amount of PA (MET-h) measured by the accelerometer used in this study is calculated in cases in which the intensity is ≥3 METs, which is equivalent to moderate-intensity PA.

The amount of PA (MET-h) data used in this study were as follows: the amount of walking activity (walking), daily activities (housework and child-rearing), and walking activity + daily activities (sum of the prior two categories). The number of steps (steps) and activity duration in METs based on the categories were also recorded.

### Statistical analysis

Basic statistics for participant characteristics and GHQ-28 were analyzed. Categorical variables were represented as percentages, whereas continuous variables were represented as means ± standard deviation (SD). The relationship between the number of childbirths (primiparous vs. multiparous) and PA duration based on intensity after categorization as well as the relationship between the GHQ-28 and amount of PA were analyzed using Welch’s *t*-test.

Welch’s *t*-test was performed after testing for homogeneity of variance and normality using Levene’s test and Shapiro–Wilk test, respectively. Linear regression analysis was also performed to elucidate the relationship between the GHQ-28 and the amount of daily activities in terms of housework and child-rearing. The dependent variable was the *amount of daily activities*, whereas the explanatory variables were the scores on the four subscales of the GHQ-28 (i.e., physical condition, anxiety and insomnia, obstacles to social activity, and depression) and the GHQ total score. Simple linear regression analysis was performed and the regression coefficient, along with its 95% confidence interval (CI) and *p* value, was calculated. Variables that were considered to affect both the *amount of daily activities* and *GHQ-28* and variables that were considered to affect either the *amount of daily activities* or *GHQ-28* were recognized as explanatory variables during multiple regression analysis using the forced entry method [40]. The adjusted variables that were entered were the *time of activities of* ≥ *3 METs, amount of walking activity, age, number of family members, nuclear vs. joint family (dummy variable),* and *total home area.* The regression coefficient, along with its 95% CI and *p* value, was calculated. All statistical analyses were performed using IBM SPSS Statistics 25 (IBM Ltd., Japan). The statistical significance was set at 5%.

### Ethical considerations

This study was approved by the Ethical Review Committee of Teikyo University of Science (approval number: 16029). All study procedures were in accordance with the Declaration of Helsinki. Written informed consent was obtained from all participants before participation in the study. The participants were informed that their participation would be of their own free will and that they could withdraw from the study at any time without suffering any disadvantage. They were also explained that the data obtained in this study would not be used for any purpose other than this study and that the study results would be made public only after anonymization to protect their privacy.

## Results

### Participant characteristics

In this study, 110 women were approached for participation, among whom 100 participated in the study. Consequently, 99 women, excluding 1 who was unable to wear the accelerometer for the required 8 h, were assessed (effective response rate, 90.0%). The mean amount of time for which the accelerometers were worn was 12.55 h. Compared with primiparous women, multiparous women were found to be engaged in greater amounts of PA as measured based on the amount of daily activities, amount of walking + daily activities, the number of steps, and energy expenditure, after excluding the amount of walking (an index of the amount of PA). Large differences were noted between the minimum and maximum values for each of the abovementioned parameters.

Compared with primiparous women, multiparous women scored higher on all four of the GHQ-28 subscales, yielding a higher mean total score, indicating the poor health status of the latter group. There were 20 multiparous women among the 30 participants whose mean total score was ≥ 6 (30.3%; Table 1).

**Table 1:** Participant characteristics

				Overall (*n* = 99)		Primiparous (*n* = 46)		Multiparous (*n* = 53)	
	Category	< Primiparous/Multiparous >	*n* (%)	Mean ± SD	Min–max	Mean ± SD	Min–max	Mean ± SD	Min–max
Age	Age category								
	20–24		3 (3.03)						
	25–29		19 (19.19)						
	30–34		51 (51.52)						
	35–39		25 (25.25)						
	40		1 (1.01)						
	Mean age			32.14 ± 3.82	21–40	30.76 ± 3.92	21–39	33.43 ± 3.24	28–40
Family unit	Nuclear		84 (84.8)						
	Joint		15 (15.2)						
Physical activity	Amount of walking (METs-h/day)			1.35 ± 1.05	0.08–6.07	1.46 ± 1.22	0.11–6.07	1.28 ± 0.89	0.08–3.73
	Amount of daily activities (METs-h/day)			3.21 ± 1.14	1.13–5.97	2.83 ± 0.92	1.13–5.00	3.39 ± 1.08	1.63–5.97
	Amount of walking + amount of daily activities (METs-h/day)			4.61 ± 1.86	1.86–9.63	4.27 ± 1.66	1.86–9.63	4.67 ± 1.46	1.91–7.40
	No. of steps (steps)			5,792.03 ± 3,207.44	1,067–17,156.0	5,368.24 ± 3,074.27	1,067.0–17,156.0	5,973.68 ± 2,555.62	1,670.0–15,736.0
	Walking time (m)			81.45 ± 35.59	3–193	73.70 ± 33.72	22.5–172.0	85.99 ± 29.40	34–154
Energy expenditure	Amount of walking + amount of daily activities (Kcal)			1966.58 ± 58	1498.5–2439.0	1930.11 ± 206.36	1498.5–2439.0	1998.22 ± 177.92	1535.0–2392.0
Body mass index	BMI = kg/m^2^			21.04 ± 2.03	16.03–26.22	21.14 ± 1.84	17.75–26.22	20.96 ± 2.20	16.03–26.22
GHQ-28	Somatic symptoms			1.62 ± 1.66	0–7	1.26 ± 1.42	0–6	1.92 ± 1.81	0–7
	Unhealthy (4 points/7 or above)	14 < 4/10 >							
	Anxiety and insomnia			1.97 ± 1.73	0–7	1.67 ± 1.79	0–7	2.23 ± 1.65	0–5
	Unhealthy (4 points/7 or above)	18 < 4/14 >							
	Social dysfunction			0.80 ± 1.33	0–7	0.70 ± 1.23	0–4	0.89 ± 1.41	0–7
	Unhealthy (3 points/7 or above)	11 < 5/6 >							
	Depression			0.12 ± 0.48	0–3	0.07 ± 0.25	0–1	0.17 ± 0.61	0–3
	Unhealthy (3 points/7 or above)	2 < 0/2 >							
	Total score			4.51 ± 4.08	0–21	3.70 ± 3.55	0–13	5.21 ± 4.40	0–21
	Unhealthy (6 points/28 or above)	30 < 10/20 >							
Total residential area	(m^2^)			67.59 ± 34.76	20.0–226.0	52.92 ± 18.11	20.0–100.0	78.79 ± 38.20	25.0–226.0

Amount of walking (METs-h/day): Value derived by multiplying PA (walking) of ≥ 3 METs with activity duration

Amount of daily activities (METs-h/day): Value derived by multiplying PA (housework and child-rearing) of ≥ 3 METs with activity duration

GHQ-28: Not at all = 0 points, not more than usual = 0 points, rather more than usual = 1 point, and much more than usual = 1 point. The scoring cutoff values were in accordance with the GHQ method. A total score of ≥ 6 points indicates mental health problems

### ***Amount of time engaged in ***PA*** based on the degree of intensity***

Light PA (lying down or sitting, including breastfeeding) corresponded to 1.0–1.9 METs. Overall, the participants engaged in this level of PA intensity for 457.64 ± 93.31 min (at least 7.5 h). The time spent doing activities of 2.0–3.9 METs, which include PAs (e.g., child-rearing and housework), was 239.65 min (4 h 39 min). Furthermore, 2.0–2.9 METs correspond to the PA intensity of light child-rearing and housework while standing. Overall, the participants engaged in this level of PA intensity for 187.67 ± 44.09 min (3 h 7 min). The activity duration was significantly longer in multiparous women than in primiparous women (*t* =  − 3.41). The participants engaged in moderate-intensity activity (at least 3 METs), which is recommended to maintain health, for 71 min/day (Table 2).

**Table 2:** Time spent engaged in activity by PA intensity (MET)

Childbirth category	Overall (min/day)	Primiparous (min/day)	Multiparous (min/day)	*t* value	*p*
PA intensity	*n* = 99	*n* = 46	*n* = 53		
1.0–1.9 METs	457.64 ± 93.31	475.43 ± 100.44	442.20 ± 84.58	1.77	0.081
2.0–2.9 METs	187.67 ± 44.09	172.12 ± 44.92	201.17 ± 38.96	− 3.41	0.001*
3.0–3.9 METs	51.98 ± 17.18	48.59 ± 19.05	54.92 ± 14.94	− 1.82	0.072
4.0–4.9 METs	14.79 ± 8.46	13.83 ± 9.43	15.62 ± 7.52	− 1.03	0.31
5.0–5.9 METs	3.21 ± 3.05	2.75 ± 2.29	3.61 ± 3.56	− 1.45	0.15
6.0–6.9 METs	0.83 ± 8.89	0.69 ± 0.63	0.95 ± 1.06	− 1.5	0.14
7.0–7.9 METs	0.14 ± 0.25	0.14 ± 0.24	0.15 ± 0.26	− 0.2	0.84
≥ 8.0 METs	0.18 ± 0.51	0.19 ± 0.69	0.18 ± 0.29	0.08	0.94

Welch’s *t*-test, *p < 0.05

1.0–1.9 METs indicates lying down or sitting (including breastfeeding)

2.0–2.9 METs indicates engaging in light child-rearing and housework while standing

≥ 3.0 METs indicates moderate PA

≥ 6.0 METs indicates vigorous PA

### Relationship between GHQ-28 and the amount of PA

The amount of PA was compared between two groups: low- and high-score groups (GHQ-28). The comparison of the low-score group, which comprised women in good health, with the high-score group, which comprised women with some type of health problem, showed that the former engaged in more PA. However, the difference was not significant. In addition, the amount of daily activities exceeded 3 METs-h/day regardless of the mental health status and total amount of daily activities—amount of walking + amount of daily activities was ≥ 4 METs-h/day (Table 3).

**Table 3:** GHQ-28 and the amount of PA

GHQ-28	Low score (*n* = 69)	High score (*n* = 30)		
Amount of PA (METs-h/day)	Mean ± SD	Mean ± SD	*t* value	*p*
No. of steps (steps)	6,022.47 ± 3,524.61	5,008.85 ± 2,380.29	1.61	0.11
Amount of walking	1.45 ± 1.16	1.12 ± 0.70	1.73	0.09
Amount of daily activities (housework and child-rearing)	3.17 ± 1.07	3.08 ± 0.96	0.42	0.68
Amount of walking + amount of daily activities	4.60 ± 1.62	4.20 ± 1.38	1.26	0.21

GHQ-28: GHQ scoring method (low score = healthy [≤ 5 points] and high score = health problems [≥ 6 points])

Amount of walking, daily activities, and walking + daily activities derived by multiplying PA of ≥ 3 METs with activity duration

Welch’s *t*-test

### Linear regression analysis of GHQ-28 and the amount of daily activities

Simple and multiple regression analyses were performed to identify whether the GHQ-28 total and subscale scores were related to the *amount of daily activities*, a subtype of the *amount of PA*. Although neither of these was significantly related to the independent variables or the *amount of daily activities*, the point estimate was negative, and the *amount of daily activities* tended to increase when the health status was good, as indicated by the low scores on the GHQ-28 subscales (Table 4).

**Table 4:** Relationship of daily activity with GHQ-28

		Simple regression analysis				Multiple regression analysis				
Dependent variables	Independent variables (GHQ-28)	Partial regression coefficient (B)	Standardized partial regression coefficient (β)	95% CI	p	Partial regression coefficient (B)	Standardized partial regression coefficient (β)	95% CI	p	VIF
	Somatic symptoms	− 0.05	− 0.17	− 0.10–0.01	0.1	− 0.04	− 0.15	− 0.09–0.00	0.05	1.09
	Anxiety and insomnia	− 0.03	− 0.10	− 0.10–0.04	0.35	− 0.01	− 0.04	− 0.06–0.04	0.61	1.04
Amount of daily activities	Social dysfunction	− 0.04	− 0.08	− 0.14–0.06	0.44	− 0.03	− 0.06	− 0.10–0.04	0.43	1.05
	Depression	− 0.04	− 0.09	− 0.13–0.05	0.36	− 0.02	− 0.05	− 0.09–0.05	0.54	1.01
	Total score	− 0.02	− 0.15	− 0.04–0.01	0.16	− 0.01	− 0.10	− 0.03–0.01	0.19	1.06

GHQ-28: Likert scale (not at all = 0 points, no more than usual = 1 point, rather more than usual = 2 points, and much more than usual = 3 points)

Dependent variable: Amount of daily activities; independent variable: GHQ-28 subscale scores and total score

Adjustment variables for multiple regression analysis (forced input): The common cause of the dependent and explanatory variables or the variable that appears to be the cause of each was used as the confounding factor. Time spent in the activities of ≥ 3 METs, walking exercise, age, number of family members (nuclear and joint families), and total home area

Amount of daily activities (METs-h/day): Value derived by multiplying PA (housework and child-rearing) of ≥ 3 METs with activity duration

## Discussion

This study evaluated PA comprising housework and child-rearing in women at 2 months postpartum to examine the relationship between PA and mental health. **A** total of 30% of the participants had mental health problems, and multiparous women had poor health. Regarding PA, many women were engaged in low-intensity activities (≤ 3 METs), but they spent substantial time on housework and child-rearing. As a result, the amount of their daily activities was at least 3 METs-h/day.

The duration of moderate or vigorous PA recommended for maintaining health is 73 min/day. In both cases, both the WHO and the Japanese standards were met. Sedentary behavior accounted for at least 7.5 h/12.5 h. The relationship between mental health and PA indicated that the amount of daily activities tended to increase when the health status was good, albeit not significantly. In addition, the amount of daily activities exceeded the recommended WHO and Japanese standards regardless of the mental health status.

### Participant characteristics

The daily mean number of steps walked by adults worldwide has been reported to be 5,000–8,000/day [41–44]. Among Japanese women (age range, 20–40 years), who walk a relatively greater number of steps than women in other countries, this value has been reported to be 6,755 steps, indicating that the number of steps walked by the participants in this study, who required time to nurse and rest, was slightly lower than the mean [44, 45].

Walking activity was calculated based on the number of steps walked by the participants using a conversion table [46]. Accordingly, the walking activity was estimated to be approximately 3 METs-h/day. However, the walking activity estimated using the accelerometer was lower (1.35 METs-h/day). Such differences may be explained by the fact that the accelerometer used in this study recorded PA with an intensity of ≥ 3 METs when a person walked at a constant speed.

These results suggested that women engage in irregular and light walking associated with child-rearing and housework during the postpartum period.

The GHQ total score was similar to that of women at 4 months postpartum [47]. The cases of health problems accounted for 30% of the total, corroborating the results reported by previous studies focusing on women at 2 months postpartum [48–51]. The mean body mass index of Japanese women at 2–4 months postpartum is 20.1 kg/m^2^ [52], which indicates that most Japanese women do not require a particular form of PA to lose weight after childbirth.

### PA

The mean sedentary time (lying down or sitting) during which the device was worn was at least 7.5 h/12.5 h. Previous studies using accelerometers reported that women at 3 months postpartum spent 8.6 h/13 h [53] and 9.3 h/19 h sitting [14]; these results were consistent with those of the present study. The time these women spent doing sedentary activities included the time spent nursing. In their study on nursing women at 2 months postpartum, Maehara et al. reported that the mean nursing frequency was 8.9 times/day and the mean nursing time was 36 min/time, indicating that at least 5 h/day was spent (sedentary) nursing, including night-time nursing [54]. The risk of venous thromboembolism is high due to increased coagulation capacity and changes in the vascular endothelium at 12 weeks after childbirth [55–57]. Hence, women in their postpartum period, particularly those who underwent cesarean section and those with obesity, should be wary of venous thromboembolism. The WHO recommends that women in their postpartum period increase the amount of PA that they engage in to any level of intensity as *doing some PA is better than doing none* [1].

In addition to hydration, it is important for women who are at risk of developing venous thromboembolism to avoid holding the same posture for a long duration during lactating.

The WHO recommends that women engage in at least 150 min/week of moderate (3–6 METs) aerobic exercise and perform activities that strengthen muscles while simultaneously engaging in low-intensity activities of daily living. Housework is also recommended as an important form of PA [1]. The results of the present study showed that the amount of time corresponding to at least 3 METs of PA exceeded 70 min/day, which can be expected to exceed 490 min/week; therefore, it exceeded the standard recommended by the WHO.

In addition, the Japanese standard for PA is stricter than the WHO standard for PA and the Japanese guideline states that at least 3 METs of PA is equivalent to 23 METs-h/week or 60 min of brisk walking every day [47].

In the present study, *the amount of daily activities* (housework and child-rearing) was equal to approximately 3 METs-h/day, which nearly met the Japanese standard. Although the participants spent a significant amount of time remaining sedentary, the results showed that the amount of their PA was adequate.

A study using accelerometers reported that women at 6 months postpartum spent > 80 min/day doing moderate to vigorous PA [7], which is in line with the results of the present study, although the study periods in the two studies differed. Evenson et al. studied women at 3 months postpartum and reported different results as they used two cutoff points. The cutoff points for PA established by Troiano et al. were 17 and 1 min/day for *moderate* and *vigorous* PA, respectively, whereas those established by Swartz et al. were 276 and 2 min/day for *moderate* and *vigorous* PA, respectively [14]. The outcomes obtained using accelerometers are difficult to compare as the cutoff points, measurement scales, and data used may differ.

Ensuring that the reproductive organs, which changed during childbirth, return to their normal functions through PA in the form of housework and child-rearing alone is difficult, although the women in the present study engaged in adequate PA. The behaviors that these women frequently engage in (e.g., motion of leaning forward and picking up an infant by one’s arms) and postures in which they lift and hold heavy objects may increase abdominal pressure [58, 59] and prevent the recovery of the pelvic floor muscles. The training of the pelvic floor and rectus abdominus muscles is important and effective in preventing stress urinary incontinence and recovering sexual function [60–62]. Thus, advising women in their postpartum period to include muscle-strengthening activities in their daily activities is necessary [1].

### Relationship between mental health and the amount of PA

The relationship between mental health and the amount of PA was not significant in this study; however, the amount of PA tended to increase when the mental health was good. Similar results were reported by McLearn et al., who showed that mothers with depression engaged in fewer activities of daily living (e.g., showing their children picture books and playing with their children) [63]. Mothers with favorable mental health tended to actively participate in child-rearing and housework. Particular attention should be paid to the amount of daily activities exceeding the levels recommended by the Japanese guidelines and WHO even in mothers who had mental health problems. Moreover, 70% of the participants in the group with health problems were multiparous women and can be assumed to engage in housework and child-rearing despite being busy and in poor physical condition. Furthermore, at least 85% of the PA performed per week by women at 3 months postpartum comprised housework and caregiving [64]; thus, women in their postpartum period perform a substantial amount of PA even when sedentary time is included.

### Study strengths, limitations, and future prospects

The results of this study are particularly important as they show that PA performed by women in their postpartum period, as measured using accelerometers, satisfies the standards set by the WHO for the amount of daily activities comprising housework and child-rearing. Therefore, these results may be highly useful in postpartum health education in the future. Specifically, the author believes that postpartum women can meet their required PA by incorporating pelvic floor and rectus abdominus muscle training to their normal daily activities. However, this is only limited to women who are not in need of postpartum weight reduction. Further, the amount of daily activities tended to increase in women with favorable mental health after child birth. Such findings will assist in conducting future confirmatory studies.

However, this study has several limitations due to the small sample size. First, the results of this study were not representative of the population. Second, a statistically significant association between the amount of daily activities and mental health could not be demonstrated because it was difficult to find information on sample size determination from previous studies.

To determine an appropriate sample size, power analysis should be performed in future confirmatory studies based on the results of this study as scientific evidence. This exploratory study revealed that many postpartum PAs were low-intensity. Accumulating evidence has shown that low-intensity PA contributes to human health. A confirmatory study that uses postpartum daily activities as the primary endpoint is warranted.

## Conclusions

The amount of daily activities (measured using accelerometers) tended to increase when mental health was good, although no significant relationship between the two was observed. Sedentary time was longer in postpartum women; however, the amount of their daily activities exceeded the standard recommended by the WHO, regardless of the mental health status. Mental health support is required for women who gave birth recently. Furthermore, educational support is needed so that they can incorporate strength training into their daily activities.

## Data Availability

The datasets generated and/or analyzed during the current study are not publicly available due to contracts with research participants but are available from the corresponding author on reasonable request.

## Abbreviations

CI: Confidence Interval
GHQ-28: General Health Questionnaire-28
MET: Metabolic Equivalent of Task
PA: Physical Activity
SD: Standard Deviation
WHO: World Health Organization
